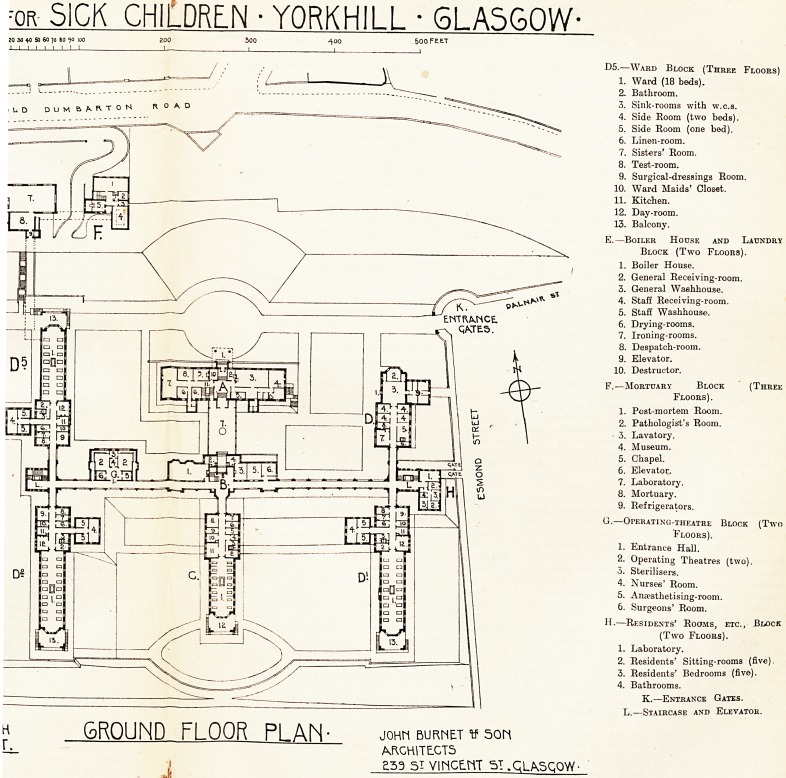# Ground Floor Plan

**Published:** 1914-07-04

**Authors:** 


### ROYAL HOSPITAL FOR SICK CHILDREN · YORK

### GENERAL PLAN REFERENCES.

A.—Administration Block and Nurses' Home (Five Floors).

1. Main Entrance.
2. Office.
3. Board Room.
4. Matron's House.
5. Matron's Business Room.
6. Home Sister's Room.
7. Nurses' Drawing-room.
8. Nurses' Writing-room.
9. Sisters' Sitting-room.
10. Visitors' Room.
11. Staircase and Elevator.

B.—Kitchen, etc., Block (Four Floors).

1. Nurses' Dining-room.
2. Pantry.
3. Residents' Dining-room.
4. Pantry.
5. Milk-preparation Room.
6. Dietetic Kitchen.
7. Conservatory.
8. Staircase and Elevator.

C.—Ward Block for Nurslings (Two Floors).

1. Ward (14 beds).
2. Bathroom.
3. Sink-room with w.c.s.
4. Nursing Mothers' Lavatory.
5. Linen-room.
6. Sisters' Room.
7. Test-room.
8. Nursing Mothers' Ward.
9. Ward Maids' Closet.
10. Kitchen.
11. Surgical-dressings Room.
12. Balcony.

D.—Admission Block (Two Floors).

1. Entrance.
2. Porters' Office.
3. Waiting Hall.
4. Examination-rooms.
5. Washing-rooms.
6. Disinfecting-room.
7. Patients' Clothes Store.
8. Nurses' Room.
9. Porter's House.

D1.—Ward Block (Two Floors).
D2.—Ward Block (Three Floors).

DONALD J. MACKINTOSH

HOSPITAL CONSULTANT.

GROUND FLOOR PLAN

### FOR SICK CHILDREN · YORKHILL · GLASGOW ·

D5.—Ward Block (Three Floors)

1. Ward (18 beds).
2. Bathroom.
3. Sink-rooms with w.c.s.
4. Side Room (two beds).
5. Side Room (one bed).
6. Linen-room.
7. Sisters' Room.
8. Test-room.
9. Surgical-dressings Room.
10. Ward Maids' Closet.
11. Kitchen.
12. Day-room.
13. Balcony.

E.—Boiler House and Laundry Block (Two Floors).

1. Boiler House.
2. General Receiving-room.
3. General Washhouse.
4. Staff Receiving-room.
5. Staff Washhouse.
6. Drying-rooms.
7. Ironing-rooms.
8. Despatch-room.
9. Elevator.
10. Destructor.

F.—Mortuary Block (Three Floors).

1. Post-mortem Room.
2. Pathologist's Room.
3. Lavatory.
4. Museum.
5. Chapel.
6. Elevator.
7. Laboratory.
8. Mortuary.
9. Refrigerators.

G.—Operating-theatre Block (Two Floors).

1. Entrance Hall.
2. Operating Theatres (two).
3. Sterilisers.
4. Nurses' Room.
5. Anæsthetising-room.
6. Surgeons' Room.

H.—Residents' Rooms, etc., Block (Two Floors).

1. Laboratory.
2. Residents' Sitting-rooms (five).
3. Residents' Bedrooms (five).
4. Bathrooms.

K.—Entrance Gates.

L.—Staircase and Elevator.

H
T.

## Figures and Tables

**Figure f1:**
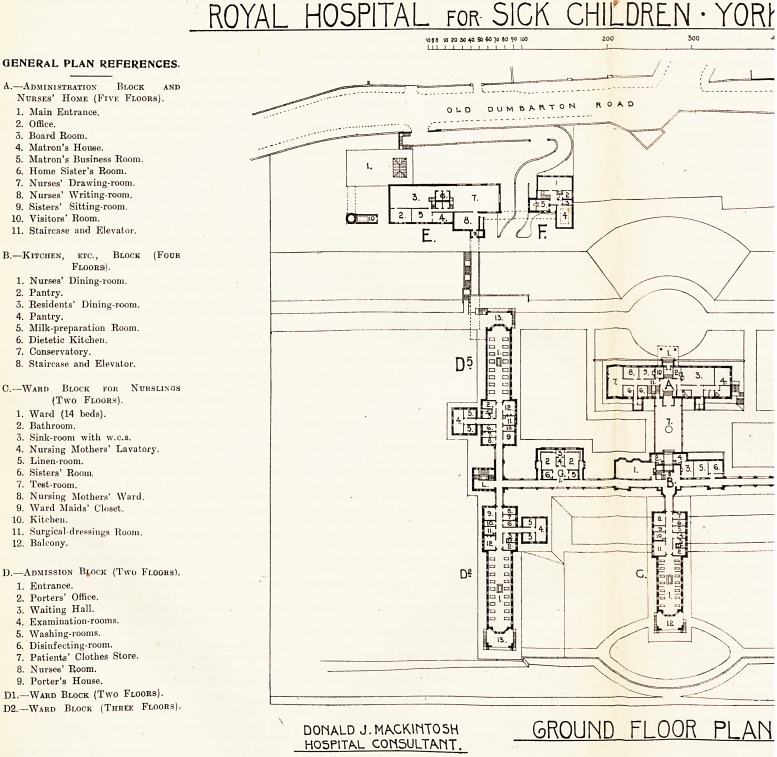


**Figure f2:**